# Radial shock waves prevent growth retardation caused by the clinically used drug vismodegib in ex vivo cultured bones

**DOI:** 10.1038/s41598-020-69904-0

**Published:** 2020-08-07

**Authors:** Sowmya Ramesh, Lars Sävendahl, Vrisha Madhuri, Farasat Zaman

**Affiliations:** 1 Department of Paediatric Orthopaedics, Christian Medical College, Vellore, 632 004 India; 2 Division of Paediatric Endocrinology, Department of Women’s and Children’s Health, Karolinska Institutet, 171 76 Solna, Sweden; 3 Centre for Stem Cell Research, A Unit of inStem Bengaluru, Christian Medical College, Bagayam, Vellore, 632 002 India; 4 Paediatric Endocrinology and Metabolism, Astrid Lindgren Children′s Hospital, Karolinska University Hospital, 171 76 Solna, Sweden

**Keywords:** Cancer, Bone, Biotechnology, Cancer, Endocrinology, Medical research

## Abstract

In childhood medulloblastoma patients, the hedgehog antagonist vismodegib is an effective anti-cancer treatment but unfortunately induces irreversible growth arrests and growth impairment limiting its use in skeletally immature patients. We hypothesized that radial shock wave treatment (rSWT) may protect drug-induced growth impairment owing to its osteogenic effects. Fetal rat metatarsal bones were exposed to vismodegib (day 0–5; 100 nM) and/or rSWT (single session); other bones from day 1 were continuously exposed to a Gli1 antagonist (GANT61; 10 µM) and/or rSWT (single session). Control bones were untreated. The bone length was measured at intervals; histomorphometric analysis and immunostaining for PCNA, Gli1, and Ihh were performed on the sectioned bones. Bones treated with vismodegib showed impaired bone growth, reduced height of the resting-proliferative zone and reduced hypertrophic cell size compared to control. In vismodegib treated bones, a single session of rSWT partially rescued bone growth, increased the growth velocity, hypertrophic cell size, and restored growth plate morphology. Bones exposed to GANT61 showed impaired bone growth and disorganized growth plate while when combined with rSWT these effects were partially prevented. Locally applied rSWT had a chondroprotective effect in rat metatarsal bones and suggest a novel strategy to prevent growth impairment caused by vismodegib.

## Introduction

Hedgehog (Hh) proteins are well-known to be overexpressed in paediatric medulloblastoma^1^. Mutations that occur in the family of Hh-pathway genes such as patched-1, suppressor of fuse and smoothened leads to an increased level of the glioma-associated oncogene (Gli1), a downstream transcription factor of Hh^2^. In the clinic, hedgehog inhibitors are used to decrease the Hh-activity and thereby impede tumor progression^3–5^. However, stable expression of the Hh-gene is essential to maintain chondrocyte proliferation and hypertrophy during bone growth^6^. A recent study reported that prolonged exposure to vismodegib, a Hh-antagonist, in children with medulloblastoma resulted in irreversible growth plate fusion causing growth arrest of long bones^7,8^. Preclinical studies in young mice exposed to a Hh-antagonist also showed growth arrests and bone growth defects^9^. Mechanistic studies revealed that even brief exposure to a Hh-inhibitor was enough to damage the growth plates by diminishing the numbers of reserve and proliferative chondrocytes^9^. These findings further imply that it may not be possible to arrive at a dose that selectively targets tumor growth with no side-effects on bone development. Therefore, in children, a protective strategy for growth plate shielding without interfering with the desired anti-cancer effects of vismodegib in the neural tissue is highly desired.

In vitro studies using cultured rat metatarsal bones^10^ and in vivo studies in rabbits^11^ and humans^12^ have shown that radial shock wave treatment (rSWT), a non-invasive modality used in the clinic, have stimulatory effects on bone growth^10^. Furthermore, the observed stimulation of chondrocyte proliferation and hypertrophy induced by rSWT was partially linked to local upregulation of Gli1 in cultured metatarsal bones^10^. Interestingly, previous in vitro studies revealed that high-energy shock wave treatment increased the uptake of chemotherapy agents^13^, thereby lowering the dose of the drug when applied to cell lines derived from human osteosarcoma^14^, human colorectal adenocarcinoma^15^, and anaplastic thyroid cancer^16^. The effect was mediated by a transient increase in cell membrane permeability allowing passage of a higher concentration of the drug^16^.

A recent report suggested that locally applied rSWT can promote longitudinal bone growth of rat metatarsal bones cultured ex-vivo in the absence of serum/growth factors^10^. Based on these findings, we hypothesized that rSWT may also have the capacity to prevent growth failure caused by Hh-inhibitors. We aimed to investigate the potential for rSWT to prevent growth retardation caused by two different Hh-antagonists, vismodegib and the Gli-1 antagonist GANT61, in a well-established model of cultured fetal rat metatarsal bones.

## Results

### Effect of vismodegib on bone growth and rescuing effects of rSWT

**Figure 1:**
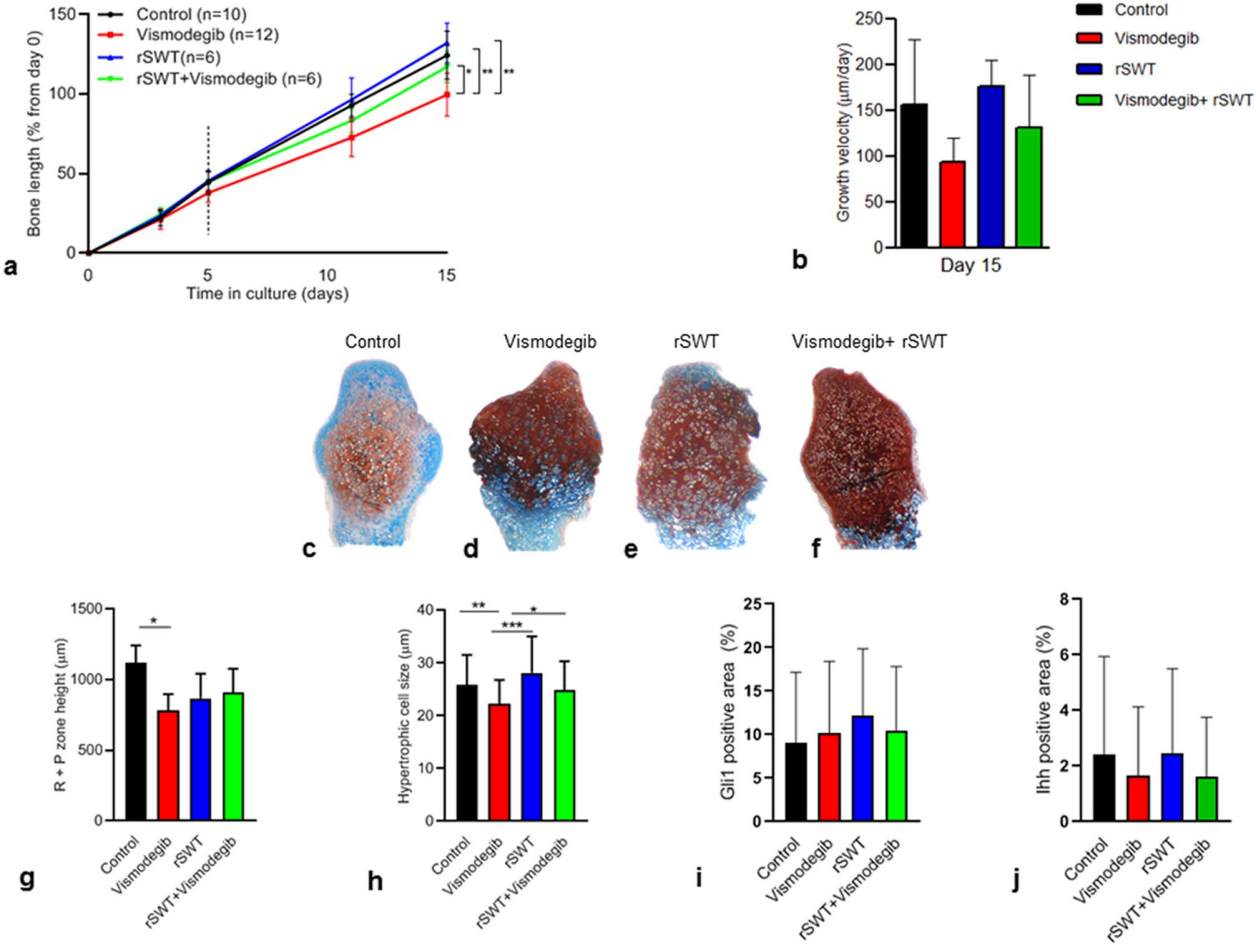
Rescuing effects of rSWT on vismodegib treated bones (**a**) Fetal rat metatarsal bones cultured ex-vivo were treated with the Hh-inhibitor vismodegib (100 nM) for 5 days (n = 12; dotted line), a single session of high-energy rSWT (500, 10 Hz, 180 mJ; n = 6), or both (n = 6), and thereafter followed for 15 days. The graph shows increases in bone length over time from day 0 (%). All error bars indicate SD. Two-way ANOVA was applied. (**b**) Graph shows the increase in the growth velocity on day 15 of control, vismodegib, rSWT, and vismodegib + rSWT treated bones. All error bars indicate SD. Representative images of metatarsal bones stained with Alcian blue; (**c**) untreated control, (**d**) vismodegib, (**e**) rSWT and (**f**) vismodegib + rSWT. Magnification 10x. (**g**) height measurements of R + P zone and (**h**) hypertrophic cell size. Quantification of immunostaining for (**i**) Gli-1 and (**j**) Ihh using the ImageJ software. *p < 0.05, **p < 0.01, ***p < 0.001.

To allow transient inhibition of Hh-activity, cultured fetal rat metatarsal bones were treated with vismodegib for 5 days whereafter growth was monitored for another 10 days without any treatment. Bones treated with vismodegib grew less than untreated controls and the difference was significant when measured on day 15 (100 ± 14% vs. 124 ± 15% bone length increase from day 0, respectively; *p* = 0.002; Fig. 1a).

To address the primary objective of this study, if rSWT can prevent growth failure caused by hedgehog inhibition, we first studied if a single exposure to rSWT can rescue metatarsal bone growth after transient exposure to 5 days treatment with vismodegib. Indeed, bones treated with rSWT (single exposure on day 0) in combination with vismodegib (day 0–5) grew significantly better than bones treated with vismodegib alone when measured on day 15 (117 ± 10% vs. 100 ± 14%, respectively; *p* = 0.02; Fig. 1a). Also, the growth velocity tended to be higher in rSWT + vismodegib compared to vismodegib alone (Fig. 1b).

### Growth plate morphology

Histological evaluation revealed severe disruption in the growth plate morphology in the bones treated with vismodegib compared to control (Fig. 1c–d). The disrupted growth plate morphology in vismodegib treated bones was found to be more normal in bones receiving rSWT alone (Fig. 1e) and rSWT + vismodegib (Fig. 1f). When comparing vismodegib treated bones to controls, we found reduced height of the resting + proliferative (R + P) zone (782 ± 117 µm vs. 1119 ± 123 µm, respectively; *p* = 0.01; Fig. 1g). The height of the R + P zone tended to be increased in bones exposed to rSWT + vismodegib when compared to vismodegib alone (910 ± 169 µm vs. 782 ± 117 µm, respectively; *p* = ns; Fig. 1g). Histomorphometric analysis showed decreased size of hypertrophic chondrocytes in the bones treated with vismodegib compared to control (22 ± 5 µm vs. 26 ± 6 µm, respectively; *p* = 0.001; Fig. 1h). In bones treated with rSWT + vismodegib, the hypertrophic chondrocytes were significantly larger compared to vismodegib alone and similar in size as in untreated control bones (25 ± 5 µm, 22 ± 5 µm, and 26 ± 6 µm, respectively; *p* = 0.02, vs. vismodegib; *p* = ns vs. control; Fig. 1h). Immunostaining for Gli (Fig. 1i) and Ihh (Fig. 1j) did not show any significant effects of vismodegib and/or rSWT.

### Shock wave treatment prevents bone growth retardation caused by GANT61

**Figure 2:**
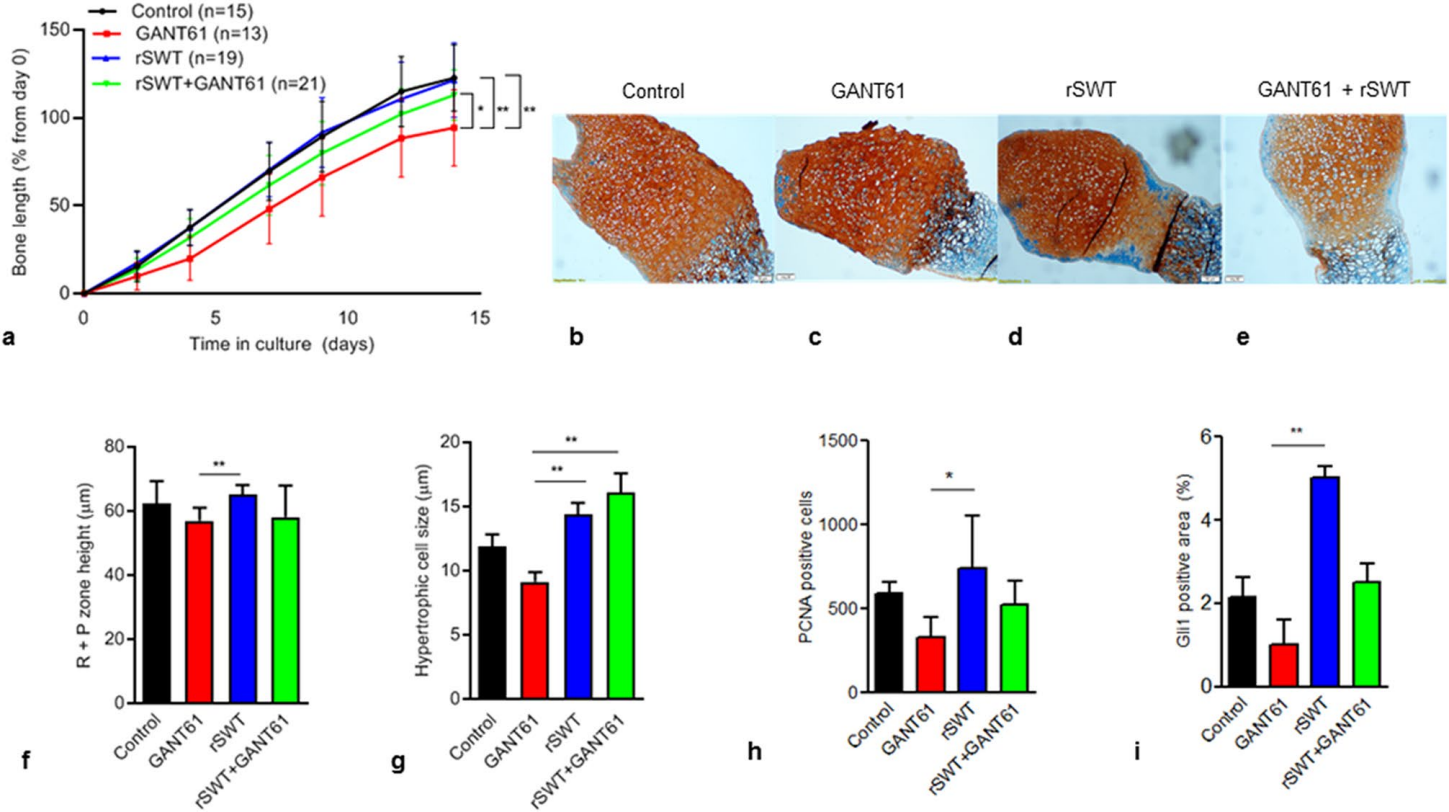
Rescuing effects of rSWT on GANT61 treated bones (**a**) Fetal rat metatarsal bones cultured ex-vivo treated with the Hh-inhibitor GANT61 (10 µM; n = 13), a single session of rSWT (500, 10 Hz, 180 mJ; n = 19), or both (n = 21), and followed for 14 days. The graph shows increases in bone length from day 0 (%). All error bars indicate SD. Two-way ANOVA was applied. Representative images of metatarsal growth plates stained with Alcian blue; (**b**) untreated control, (**c**) GANT61, (**d**) rSWT and (**e**) GANT61 + rSWT. Magnification 10x. (**f**) Height measurements of R + P zone and (g) hypertrophic cell size. Quantification of immunostaining for (**h**) PCNA and (**i**) Gli-1 using the ImageJ software. *p < 0.05, **p < 0.01.

To study the effects of continuous inhibition of Hh-activity, fetal rat metatarsal bones were treated with the Gli-1 antagonist GANT61 from day 1 of culture until the termination of the experiment on day 14. Already on day 4, GANT61 induced a pronounced suppression of the bone growth (20 ± 12%, 38 ± 10%; *p* = 0.001, vs. control) which remained at endpoint on day 14 (94 ± 22%, 123 ± 19%; *p* = 0.003, vs. control; Fig. 2a).

Next, we asked if a single exposure to rSWT can rescue from growth retardation caused by continuous Hh-inhibition induced by GANT61. Indeed, metatarsal bones exposed to rSWT on day 1 were rescued from GANT61-induced growth retardation already from day 4 of culture (32 ± 11%, 20 ± 12%; *p* = 0.02 vs. GANT61 alone) which remained until endpoint (113 ± 15%, 94 ± 22%; *p* = 0.03 vs. GANT61 alone; Fig. 2a).

### Growth plate morphology

Compared to control, in the bones continuously treated with GANT61, growth plate morphology was found to be disturbed with disorganized chondrocyte columns on day 14 (Fig. 2b, c). Bones treated with rSWT alone showed organized growth plate morphology (Fig. 2d). The growth rescuing effect of rSWT in GANT61 treated bones was accompanied by a normalization of growth plate morphology including organized chondrocyte columns (Fig. 2e).

Histomorphometric analyses showed no significant differences in the height of the R + P zone between GANT61 treated bones with or without rSWT (58 ± 9 µm, 57 ± 4 µm; *p* = ns; Fig. 2f). There was an increased size of hypertrophic chondrocytes in rSWT + GANT61 group when compared to GANT61 alone (16 ± 4.2 µm, 9 ± 2 µm; *p* = 0.002; Fig. 2g). Furthermore, the number of proliferative chondrocytes tended to be lower in GANT61 treated bones when compared to control (330 ± 117 cells, 591 ± 67 cells; *p* = ns; Fig. 2h). The number of proliferative PCNA positive cells tended to be higher in the rSWT + GANT61 group compared to GANT61 alone (524 ± 144 cells, 117 ± 58 cells; *p* = ns; Fig. 2h). Immunoexpression of Gli1 tended to be reduced in GANT61 treated bones (*p* = ns vs. control) whereas bones treated with rSWT + GANT61 tended to have higher Gli-1 expression compared to GANT61 alone (*p* = ns) (Fig. 2i).

## Discussion

We aimed to investigate the potential for locally applied rSWT to prevent bone growth impairment caused by the hedgehog inhibitor vismodegib, a therapeutic investigational drug, using a well-established model of ex vivo cultured fetal rat metatarsal bones. Herein, we report that a single session of rSWT partially prevented growth retardation caused by both transient and continuous Hh-inhibition induced by vismodegib and GANT61, respectively. The growth rescuing effects by rSWT were accompanied by preservation of growth plate morphology disrupted by the Hh-inhibitors. Altogether, our data suggest that rSWT has the potential to non-invasively protect bones from growth retardation caused by vismodegib.

Bone growth is majorly dependent on the preservation of a unique organization of chondrocytes in the growth plate^17^. Recent reports have demonstrated that long term exposure to vismodegib, the first Hh-antagonist approved in the US by FDA, led to permanent growth impairment in children with medulloblastoma^7,8^. To date, no successful strategy that targets tumor cells with no adverse effect on longitudinal bone growth has been described. Previous reports have shown that rSWT, a treatment modality that is already used in children for musculoskeletal indications^18^, can stimulate longitudinal bone growth locally in ex-vivo cultured metatarsal bones even in the absence of any systemic growth factors^10^. Pro-inflammatory cytokines are also known to impair bone growth^19^ and interestingly shockwave treatment has been shown to reduce inflammation and apoptosis while stimulating the regeneration of various tissues^20,21^. These findings encouraged us to expand this knowledge and further investigate the potential for rSWT to prevent bone growth impairment caused by Hh-inhibitors.

Vismodegib at 100 nM concentration has shown to impair bone growth in ex-vivo cultured metatarsal bones^22^ and decrease proliferation of the precursors of cerebellar granule neurons^23^, while in vivo studies in a model of medulloblastoma have also shown that vismodegib inhibits Gli1 at a IC50 of 165 nM^24^, a similar range of concentration as used in the present study. In young mice, transient inhibition of the Hh pathway has been reported to cause permanent defects in bone and growth plate structure^9^. Similar to the previous in vivo observations in young mice^9^, our histomorphometric growth plate data suggest that partial loss of Hh-activity may result in the breakdown of chondrocyte columnar organization and reduced size of hypertrophic chondrocytes. Also, the disrupted growth plate ultrastructure caused by Hh-inhibition explains the observed growth deficit in our study model system. Besides, undesired apoptosis of stem-like cells within the growth plate is another well-known contributing factor linked to growth retardation caused by anti-cancer drugs^25,26^.

Our key finding is that a single administration of rSWT not only prevented bone growth retardation caused by transient exposure to the Hh-inhibitor vismodegib but also rescued bone growth under a condition of continuous Hh-inhibition induced by another Hh-inhibitor GANT61. Furthermore, rSWT also improved growth velocity and restored growth plate morphology in bones exposed to vismodegib or GANT61. Thus our findings highlight the potential for shock wave technology to be developed as a new and safe treatment strategy to minimize deleterious effects of Hh-inhibitors selectively in the growth plates of treated children.

Hedgehog signaling drives chondrocyte proliferation and hypertrophy in the growth plate cartilage^6^. From in vitro studies, we know that Hh-inhibitors decrease the expression of Gli1 and induce cell cycle arrest in prostate cancer cells^27^. Despite rSWT rescued bones from Hh-inhibitor impaired bone growth, we did not see significant alterations in the expression of Gli1 and Ihh suggesting a cross-talk between hedgehog signaling and other pathways^28^. We speculate that the bone rescuing effect of rSWT is more evident if there is any ongoing disturbance within growth plate chondrocytes. Indeed, it was interesting to note that despite continuous exposure to a Hh-inhibitor, a single session of rSWT could partially rescue the bone growth.

Our study has several limitations. Firstly, the bone growth rescuing effects of rSWT were documented in an ex-vivo bone culture model and we do not know if this will be applicable under in vivo conditions. Nevertheless, in vivo studies in rats or mice are of limited value when it comes to exploring the potential for rSWT to rescue from vismodegib induced bone growth impairment as their growth plates do not normally fuse^29^. Secondly, we only applied a single dose of rSWT while multiple sessions could potentially be even more efficient when it comes to preventing growth impairment caused by Hh-inhibitors. Thirdly, the concentration of vismodegib used in the present study is different from the plasma concentration (8.8 µM) achieved in children^30^. Nevertheless, mimicking a gradual decline in bone growth in order to test the rescuing effect of rSWT is more important in our experimental setting. We, therefore, claim a protective effect and not a clinical effect which will require more rigorous testing. Consequently, our proof of concept finding opens up a window of opportunity to explore the potential for locally applied rSWT to prevent bone growth impairment caused by vismodegib as it may not be possible to extrapolate the doses used for preclinical studies to a clinical setting^31,32^.

**Figure 3:**
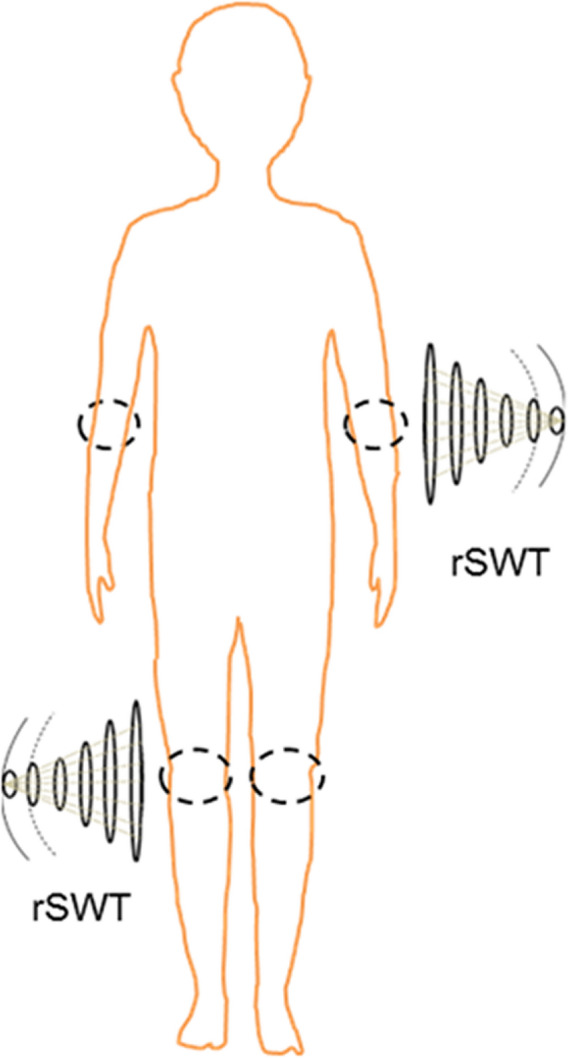
A hypothetical schematic diagram showing how extracorporeal radial shockwave treatment (rSWT) may be administered to low/high growth velocity areas (encircled) selectively to restore vismodegib-induced bone growth impairment in children with medulloblastoma.

In summary, we here present a novel treatment strategy based on clinically used rSWT to locally prevent bone growth impairment caused by vismodegib, a promising anti-cancer drug used in children with medulloblastoma (Fig. 3). Before any clinical studies, our promising ex vivo findings need to be validated in an in vivo animal model like the rabbit where growth plate fusion normally occurs, just like in humans.

## Methods

The experiments were approved by the local institutional review board (Min No. 8513) and the institutional animal ethics committee (Min No. 10/2019) at Christian Medical College, Vellore. Animal care compiled with the Guide for the Care and Use of Laboratory Animals. A radial shock wave machine from Radialspec (Medispec, Gaithersburg, MD, USA) was used for the study.

### Bone organ culture system

**Figure 4:**
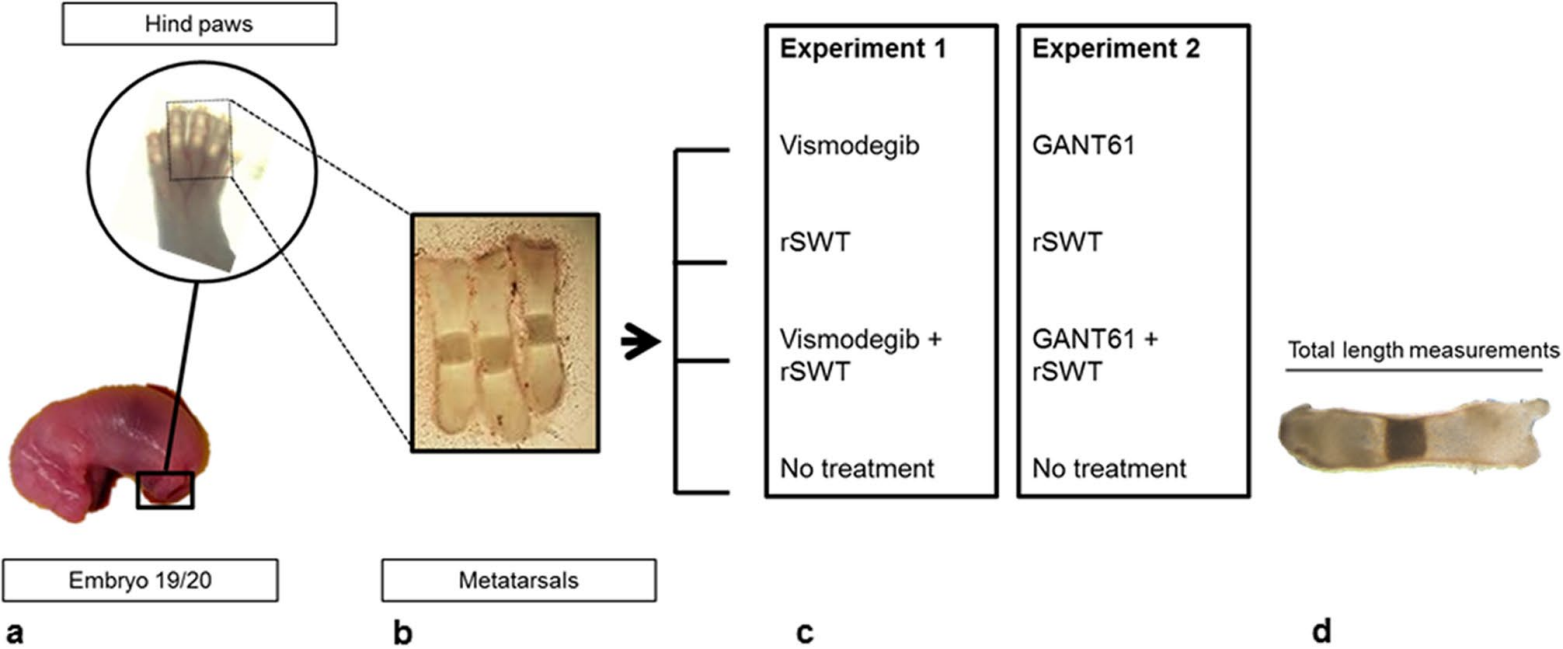
Experimental overview: Middle-three metatarsals from the hind limb of embryonic rat at (**a**) 19/20th day of gestation were dissected and (**b**) cultured ex-vivo; (**c**) Bones were either exposed to vismodegib/GANT61, a single session of high-energy radial shock wave treatment (rSWT), vismodegib/GANT61 with rSWT or left untreated. During this time (**d**) total bone length measurements at different intervals are performed.

Metatarsal bones were microdissected from the hind limbs of Sprague Dawley rat fetuses sacrificed on day 20 of gestation. Ex vivo cultures were performed as previously reported^33^. Briefly, each bone was transferred to a 24-well plate and cultured in medium containing DMEM/F12, 10 mM beta-glycerophosphate, ascorbic acid (50 µg/ml) and gentamycin. The medium was replenished every two days. Figure 4 shows the experimental overview.

#### Exposure to Vismodegib

Vismodegib was purchased from Selleck Chemicals (Houston, Texas, USA). The experimental setup consisted of four groups. Metatarsal bones were cultured for 15 days and were either (a) treated with vismodegib (100 nM; n = 12) from day 0 to 5, (b) a single exposure to rSWT (500 impulses, 10 Hz, 180 mJ; n = 6) on day 0, (c) vismodegib + rSWT (n = 6) or (d) were left untreated (control; n = 10). The bone length was measured on days 0, 3, 5, 11, and 15.

#### Exposure to GANT61

GANT61 was purchased from Sigma Aldrich (St. Louis, Missouri, USA). Metatarsal bones were treated with the (a) Hh-inhibitor GANT61 (10 µM) from day 1 to 14 (n = 13), (b) a single exposure to rSWT on day 1 (500 impulses, 10 Hz, 180 mJ; n = 19), (c) GANT61 + rSWT or (d) were left untreated (control; n = 15). The bone length was measured on days 0, 2, 4, 7, 9, 12, and 14.

### Bone length measurement

Digital images were captured for bone length measurements using an inverted microscope (Leica Microsystems). All measurements were performed by one of the investigators blinded to the nature of the group using an inbuilt ‘measurement tool’. Bone growth is expressed as percent bone length increase from day 0. Bowed bones were measured in two parts added together.

### Quantitative histology and immunostaining

After termination of the culture, the metatarsal bones were fixed with 4% paraformaldehyde, embedded in paraffin and five-micrometer sections were cut along the longitudinal axis (proximal to distal) followed by staining with Safranin-O and Alcian blue. The microscopic description of the growth plate morphology included an assessment of the organization of the chondrocyte column. Histomorphometric analysis was performed to measure the height of the resting-proliferating zone at five different regions of the growth plate and the size of hypertrophic chondrocytes. Hypertrophic cells were defined by a height along the longitudinal axis greater than 7 µm. Eight hypertrophic chondrocytes from the proximal and distal growth plate were measured.

Immunostaining was performed as previously described^34^. Antigen retrieval was performed in citrate buffer at 90° Celsius and endogenous peroxidase activity was quenched with 3% H_2_O_2_ in methanol for 10 min followed by a wash with PBS. For immunostaining, sections were blocked with 10% bovine serum albumin for 1 h, incubated with primary antibodies (1:50 dilution) Gli1 (Abcam, Cambridge, MA, USA) and Ihh mouse monoclonal antibody (Santa Cruz Biotechnology, Dallas, TX, USA) overnight at 4° Celcius. After incubation for 1 h with secondary polyclonal anti-mouse or anti-rabbit biotinylated antibody (DakoCytomation, Glostrup, Denmark, 1:500 dilution), sections were incubated with ABC solution and developed with diaminobenzidine. Sections were counterstained with Alcian blue. Non-immune immunoglobin G (IgG) of the same species as the primary antibodies were used as negative controls. Three to five bones per group were analyzed. To quantify immunostaining, the ImageJ software (National Institutes of Mental Health, Bethesda, MD, USA) was used, and the percentage of DAB positivity was calculated digitally using a plugin IHC profiler.

### Statistical analysis

All statistics were carried out using GraphPad Prism 8.0 (GraphPad Software, Inc, La Jolla, CA, USA). Data were summarized using means ± SD for the bone length measurements and histomorphometric assessments. A two-way ANOVA with Dunnett multiple comparisons test^35^ was performed to examine the change in bone length in terms of treatment and days. Pairwise comparisons were done corrected for the alpha levels. Margin plots with SD were presented to visualize the change in bone length. Kruskal–Wallis and Man–Whitney U test were performed when the data were not normally disturbed. A *p* value of < 0.05 was considered to indicate a significant difference.

## Data Availability

All data generated or analysed during this study are included in this manuscript.
